# Recognizing Pemphigus Vulgaris in a Low Prevalence Setting: A Journey Through Multiple Diagnoses

**DOI:** 10.1155/crdm/9778760

**Published:** 2026-04-30

**Authors:** Maria Emilia Adenike V. Adedoja, Ma. May Jasmin Ramos Yason, Melissa Aquino-Villamin

**Affiliations:** ^1^ Department of Internal Medicine, De Los Santos Medical Center, Quezon City, Philippines; ^2^ Section of Dermatology, De Los Santos Medical Center, Quezon City, Philippines; ^3^ Section of Rheumatology, De Los Santos Medical Center, Quezon City, Philippines

**Keywords:** autoimmune blistering disease, Behçet’s disease, diagnostic delay, oral ulcers, pemphigus vulgaris

## Abstract

Pemphigus vulgaris (PV) is an autoimmune blistering disease that may present predominantly with mucosal involvement and is frequently misdiagnosed as infectious or inflammatory conditions. We describe a 72‐year‐old Filipino woman with a 3‐month history of painful oral and genital ulcerations and subsequent flaccid bullae who was initially diagnosed and treated sequentially for candidiasis, disseminated herpes zoster infection, and Behçet’s disease based on International Criteria for Behçet’s Disease scoring. Despite multiple antimicrobial therapies, her symptoms progressed. Definitive evaluation revealed intraepidermal acantholysis on histopathology and intercellular IgG and C3 deposition in a characteristic “fishnet” pattern on direct immunofluorescence, with positive desmoglein 1 and 3 antibodies, confirming PV. Prompt initiation of systemic corticosteroids led to rapid clinical improvement. This case underscores how diagnostic anchoring and reliance on classification criteria can delay recognition of autoimmune blistering disease, highlighting the need for early biopsy and direct immunofluorescence in persistent mucocutaneous ulceration.

## 1. Introduction

Pemphigus vulgaris (PV) is an uncommon autoimmune condition characterized by blistering, primarily impacting the skin and mucous membranes throughout the body. Early diagnosis is often challenging, especially when mucosal involvement predominates, as patients may initially present with symptoms mimicking infectious or inflammatory diseases [1, 2]. This can lead to delays in diagnosis, which can lead to prolonged inappropriate therapy and increased morbidity [2, 3]. In the Philippines, where published epidemiologic data are limited and PV appears to be low prevalence, misclassification may be more likely. This report highlights a case of PV initially diagnosed as candidiasis, disseminated herpes zoster and subsequently as Behçet’s disease, emphasizing the importance of careful clinical and diagnostic evaluation.

## 2. Case Presentation

A 72‐year‐old Filipino woman with type 2 diabetes mellitus maintained on linagliptin and metformin with good compliance and with a family history of autoimmune thyroid disease, presented with a 3 month history of multiple painful ulcerations on the labia majora, lower lip, tongue, and palate with subsequent appearance of flaccid bullae on the torso and back that easily ruptured, leaving painful erosions with sparing of the palms and soles. She first sought gynecologic consultation for vulvar ulcers, where a fungal infection was initially suspected, and was given fluconazole 400 mg/tab once daily for 7 days. Owing to persistent symptoms, she was referred to an adult infectious disease specialist and was treated for disseminated varicella zoster with a secondary bacterial infection. She was prescribed acyclovir 800 mg/tab five times daily and co‐amoxiclav 625 mg/tab thrice daily for 10 days; however, no clinical improvement was observed after this treatment. Due to worsening oral pain, she restricted her intake and later experienced a presyncopal episode, prompting hospital admission.

On initial evaluation, the patient was generally weak, with a temperature of 37.9°C, swollen lips, multiple irregular ulcerations on the tongue and labial mucosa, a white plaque covering the posterior two‐thirds of the tongue, hyperemic oral mucosa, tonsils, and friable erosions with irregularly erythematous borders at the xiphoid process (Figure 1), with multiple anal fissures. Nikolsky sign was not formally assessed during the initial examination. No ocular inflammation was observed. Behçet’s disease was suspected. Her International Criteria for Behçet’s Disease (ICBD) was a total of five points (oral aphthosis, genital aphthosis, and skin lesions) [4].

FIGURE 1Clinical Findings. (a) Multiple irregular ulcerations and erosions on the dorsal tongue and labial mucosa with overlying white plaques. (b) Eroded cutaneous lesion with erythematous borders located near the xiphoid process.

**Figure:**
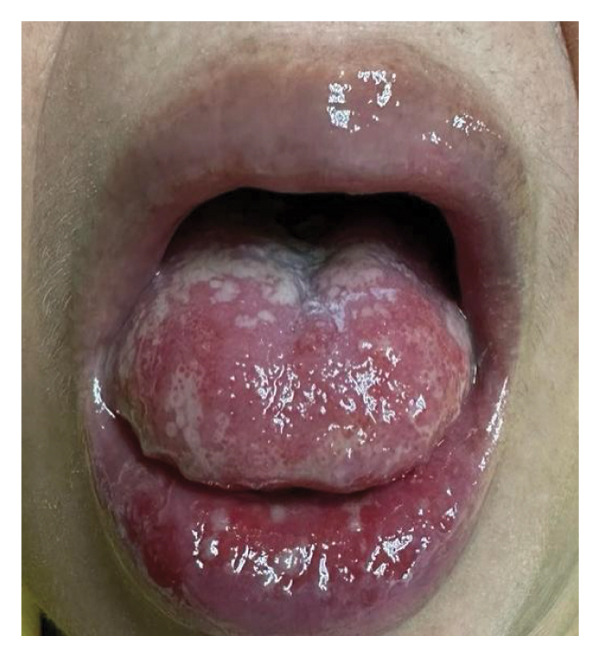
(a)

**Figure:**
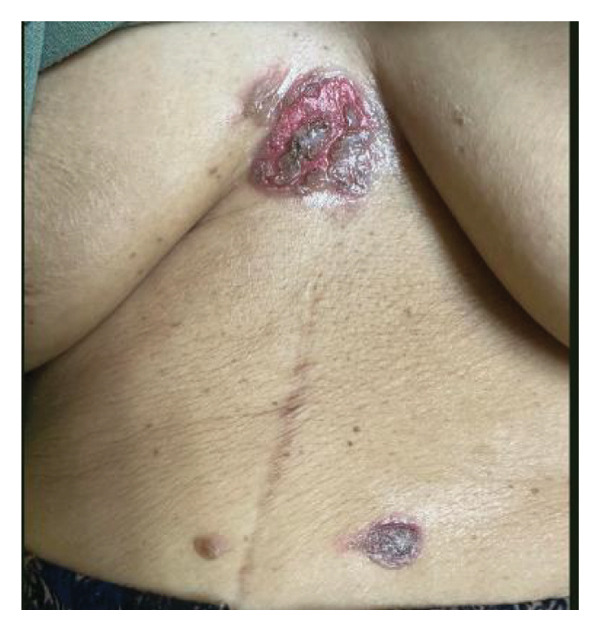
(b)

**FIGURE 2:**
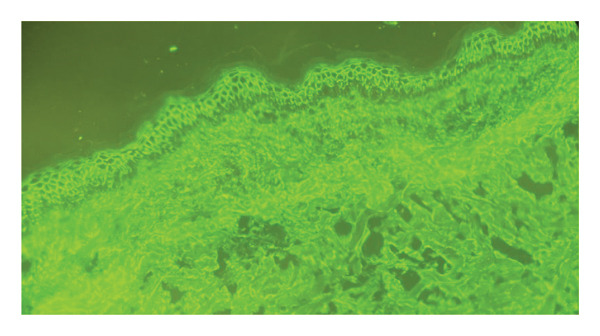
Direct immunofluorescence, original magnification x 100, reveals IgG and C3 deposits between cells, forming a distinctive “fishnet” or “chicken‐wire” pattern.

Initial laboratory workup revealed a hemoglobin level of 114 (reference range: 123–153 g/L), leukocytes of 9.21 (reference range: 4.23–10.04 10^9^/L), and platelet count of 199 (reference range 130–500 10^9^/L). Sodium (Na) was 131 mEq/L (138–146 mEq/L), potassium (K) was 4.40 mEq/L (reference range: 3.5–4.9 mEq/L), aspartate aminotransferase (AST) was 24 (reference range: 5–34 U/L), alanine aminotransferase (ALT) was 16 (reference range: 6–55 U/L), erythrocyte sedimentation rate was elevated at 24 mm/hr (reference range: 0–10 mm/hr), C‐reactive protein (CRP) was 42 mg/L (reference range: less than 6 mg/L), and blood culture was negative. The pathergy test, used for identifying Behçet’s disease, showed no typical reaction, further complicating the initial diagnosis and underscoring the need for comprehensive testing. Autoimmune serologic tests for anti‐Sjögren ‘s syndrome‐related antigen A (anti‐SSA), anti‐Sjögren ‘s syndrome‐related antigen B (anti‐SSB), and antinuclear antibody (ANA) titers were negative (Table 1), decreasing the likelihood of Sjögren syndrome or ANA‐driven connective tissue disease. Skin biopsies were advised for definitive testing. Oral and skin histopathology and direct immunofluorescence (DIF) were performed. Histopathological examination of the skin revealed intraepidermal acantholysis with a “row of tombstones,” mild perivascular lymphocytic infiltrate, dermal edema, and few eosinophils. DIF studies demonstrated intercellular deposition of IgG (faint) and C3 (intense), predominantly in the lower spinous and basal layers, displaying a characteristic “fishnet” or “chicken wire” pattern, consistent with PV (Figure 2). ELISA was positive for desmoglein 1 and 3. These findings confirmed the diagnosis of PV [5].

**TABLE 1:** Serology and initial laboratory results (with reference ranges).

Test	Reference range	Result
Anti‐Sjögren’s syndrome‐related antigen A (anti‐SSA)	Positive/negative	Negative
Anti‐Sjögren’s syndrome‐related antigen B (anti‐SSB)	Positive/negative	Negative
Antinuclear antibody (ANA)	Positive/negative	Negative
Hemoglobin	123–153 g/L	114 g/L
Leukocytes	4.23–10.04 × 10^9^/L	9.21 × 10^9^/L
Platelet count	130–500 × 10^9/^L	199 × 10^9/^L
Na	138–146 mEq/L	131 mEq/L
K	3.5–4.9 mEq/L	4.40 mEq/L
AST	5–34U/L	24 U/L
ALT	6–55U/L	16 U/L
ESR	0–10 mm/hr	24 mm/hr
CRP	Less than 6 mg/L	42 mg/L

*Note:* Na: sodium, K: potassium, AST: aspartate aminotransferase, ALT: alanine aminotransferase.

Abbreviations: CRP, C‐reactive protein; ESR, erythrocyte sedimentation rate.

The patient was started on intravenous hydrocortisone (100 mg every 8 h) plus meticulous oral care with moisturizing gels and pantoprazole 40 mg intravenously daily. One week after treatment, oral pain improved from 8/10 to 2/10 with complete re‐epithelialization, and no new lesions developed (Figure 3). The patient’s quality of life has improved. Hydrocortisone was shifted to prednisone 30 mg/tab daily, with plans to taper in the following weeks. A steroid‐sparing agent, mycophenolate mofetil (500 mg/tab), was initiated at 1 tab twice daily in line with guideline‐concordant care. Antibiotics were reserved only for proven or strongly suspected secondary infection [5]. Close follow‐up was arranged. The patient was followed for one year with regular visits every two weeks during the early treatment period. The patient demonstrated sustained clinical improvement with only minimal residual palatal lesions and no major recurrence of mucocutaneous disease.

FIGURE 3Clinical improvement following corticosteroid therapy. (a) Resolution of oral mucosal lesions with absence of aphthous ulcers on the tongue. (b) Resolution of cutaneous lesion near the xiphoid process with residual postinflammatory changes.

**Figure:**
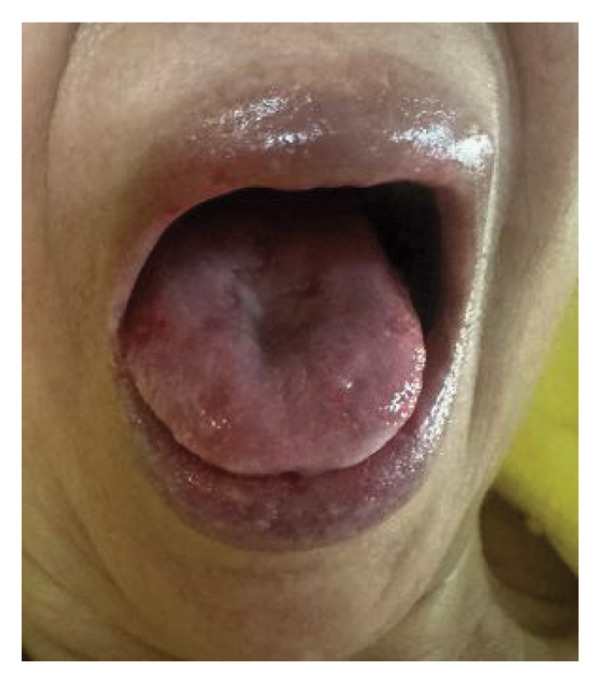
(a)

**Figure:**
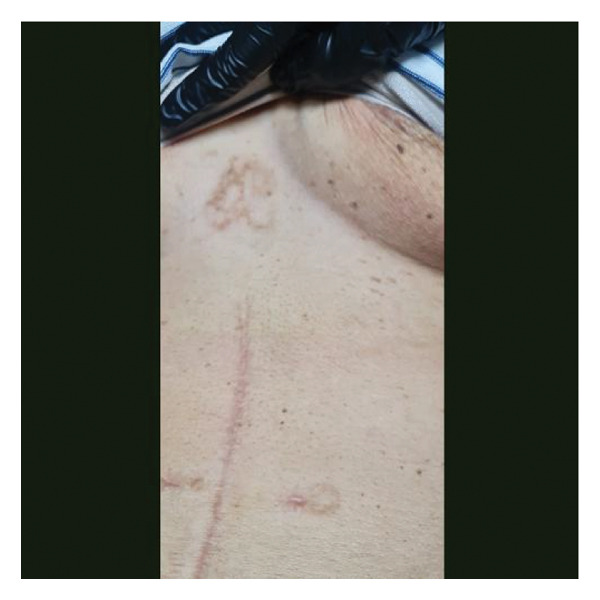
(b)

## 3. Discussion

Diagnostic delays in mucosal‐predominant PV are well documented, with studies reporting mean delays of 6 months from symptom onset to definitive diagnosis [6]. Several key clinical findings should have raised suspicion for PV rather than Bechet’s disease. Although PV and Behçet’s disease share some clinical similarities, such as oral ulcers and skin lesions, there are key differences that can help healthcare providers make an accurate diagnosis [7]. Hallmarks that favor PV include flaccid rather than indurated or aphthous lesions, a positive Nikolsky sign on perilesional mucosa where recognition of this sign should prompt immediate consideration of an autoimmune blistering disorder and expedite biopsy with DIF [8].

The ICBD criteria, developed through a multicenter international collaboration, demonstrate a sensitivity of 93.9% and specificity of 92.1% for classifying BD in research settings. Recurrent oral ± genital ulcers may yield an ICBD score at or above the classification threshold; however, the classification criteria support research uniformity and do not substitute for clinical diagnosis. Although ICBD has high sensitivity, classification criteria should be interpreted within the full clinical context, particularly when competing diagnoses with overlapping mucocutaneous features are present [4, 9]. Systemic corticosteroids remain the cornerstone of treatment for PV and are often combined with immunosuppressive agents such as mycophenolate mofetil or azathioprine to reduce steroid exposure [10, 11]. In recent years, rituximab, an anti‐CD20 monoclonal antibody, has emerged as an effective first‐line therapy for moderate to severe PV and has been shown to achieve higher rates of sustained remission compared with corticosteroid therapy alone [12].

Accordingly, an efficient work‐up prioritizes prompt perilesional biopsy and DIF in ambiguous mucosal disease to avoid anchoring on BD and to unnecessary empiric antibiotics, thereby accelerating PV‐specific management [13]. In the absence of treatment, PV exhibits a mortality rate between 60% and 90%. Additionally, it can result in numerous life‐threatening issues, such as sepsis, heart failure, and kidney failure. The introduction of systemic corticosteroids has successfully reduced the mortality rate among patients with PV to approximately 10% [14]. Early recognition and prompt corticosteroid therapy can lead to favorable clinical outcomes and significant improvements in quality of life [14].

## Funding

The authors have nothing to report.

## Consent

Written informed consent for publication of clinical information and photographs was obtained from the patient. Patient consent forms were not provided to the journal but are retained by the authors.

## Conflicts of Interest

The authors declare no conflicts of interest.

## Data Availability

The data that support the findings of this study are available on request from the corresponding author. The data are not publicly available due to privacy or ethical restrictions.
